# Editorial: Physical activity, self-regulation, and executive control across the lifespan

**DOI:** 10.3389/fnhum.2015.00614

**Published:** 2015-11-06

**Authors:** Sean P. Mullen, Peter A. Hall

**Affiliations:** ^1^Exercise Technology and Cognition Laboratory, Department of Kinesiology and Community Health, University of Illinois at Urbana-ChampaignUrbana, IL, USA; ^2^Prevention Neuroscience Lab, Faculty of Applied Health Sciences, University of WaterlooWaterloo, ON, Canada

**Keywords:** cognitive control, self-regulation, executive functioning, physical activity, fitness-cognition link, behavioral maintenance, exercise adherence, cardiorespiratory fitness

Physical activity is a complex behavior that involves iterative planning, monitoring, ongoing adjustments, and inhibition of unwanted distractions. These same processes are manifestations of executive control and rely on established neural networks involving the prefrontal cortex (see Buckley et al., 2014). A plethora of evidence exists showing that continued involvement in a physical activity program can enhance multiple domains of executive control. More recently, research has also indicated that executive functioning is associated with the long-term maintenance of physical activity participation. This Research Topic comprises theoretical perspectives (Buckley et al., 2014; Hall and Fong, 2015) and empirical findings (Anderson-Hanley et al., 2014; Best et al., 2014; Blanchfield et al., 2014; Daly et al., 2014; Leckie et al., 2014; Lowe et al., 2014; Moore et al., 2014; Dupuy et al., 2015; Pageaux et al., 2015) underlying the reciprocal relationship between physical activity and executive control.

Although the fitness-cognition link has received a great deal of attention, particularly the effect of cardiorespiratory fitness training on subsequent cognitive performance, there is still much to be learned in children and adults. In a cross-sectional study, Moore et al. (2014) extended this work by showing that children with higher fitness levels performed better on a “large problem” arithmetic task. Moreover, higher fit children showed selective modulation in electrophysiological indices (N170, P3, and N400), suggesting that fitness may augment encoding, attention, and processing during arithmetic tasks. In a similarly designed study of young and older adult women, Dupuy et al. (2015) found that, irrespective of age, fitness was positively associated with Stroop task performance, as well as higher cerebral oxygenation in the right inferior frontal gyrus. In addition to the imaging conducted in the Moore et al. (2014) and Dupuy et al. (2015) studies which offered insight into neurophysiological mechanisms contributing to fitness-enhancing effects on the brain, Leckie et al. (2014) tested a mediational model with brain-derived neurotrophic factor. Specifically, Leckie et al. found that BDNF-1 mediated the effect of a 1-year walking intervention on task-switching performance, but only for those 71 and older. Together, these findings suggest that certain patterns of electrophysiological activity and levels of oxygenated blood in the prefrontal cortex are associated with better fitness levels and executive performance, in children and adults, respectively; whereas physical activity-induced neurotransmitter release may serve to protect brain health among the oldest adults.

It is well-known that one must remain physically active for 6 months or longer to attain a high level of cardiorespiratory fitness. Yet it is also well-understood that many individuals are incapable of sustaining a physical activity program for this long on their own. Rather, successful maintenance of physical typically requires substantial support and supervision. Even then, a high percentage of people drop out from their programs simply due to difficulties negotiating everyday costs of activity participation (e.g., scheduling conflicts, resisting competing sedentary activities). From this perspective, although exercise itself may have benefits for the brain centers that support executive control, it may also be the case that strong executive functioning may itself facilitate consistency for this challenging activity. Two studies in this section support this hypothesis. In the first, Daly et al. (2014) tested a bidirectional relationship between physical activity and executive control (defined by verbal fluency and a letter cancellation task), using a prospective design and a large (*n* = 4555) nationally-representative older adult sample. They found poor executive control was associated with lower self-reported physical activity rates over a 2-year period. Moreover, executive control's contribution to physical activity was 50% greater in magnitude than the contribution of physical activity to subsequent changes in executive control. In a second study, examining data from a randomized controlled trial (RCT) with 125 older women, Best et al. (2014) tested the effect of strength training-induced improvements in executive control on subsequent self-reported physical activity during an unsupported, 1-year follow-up period. They found that greater training-induced Stroop gains were associated with better adherence during the unstructured follow-up period.

The effects of aerobic exercise-induced changes in executive control on subsequent maintenance of physical activity may not be entirely straightforward, however. Anderson-Hanley et al. (2014) reported a 6-month follow-up to an RCT involving older adults (40% meeting criteria for mild cognitive impairment), who engaged in 3 months of virtual-reality-enhanced aerobic cycling. Specifically, those with poorer executive control were more likely to engage in self-regulated cycling during the observational period following their supervised program. This inverse relationship was unexpected. The researchers suggested that this population might have been especially committed to counteract their own decline in cognitive functioning. It is also important to note that no other types of physical activity were assessed during the “naturalistic window” and participants may have disengaged from other lifestyle activities such as walking.

For several decades, researchers have also reported evidence of acute effects of exercise on cognitive functioning. Little is known about the direct and indirect spillover effects that exercise and the exercising of one's self-regulatory control may have on other areas of life. Interestingly, Lowe et al. (2014) showed that involvement in a single bout of moderate intensity aerobic activity increased Stroop task performance, and that those with larger effects consumed more non-appetitive foods, possibly representing a compensatory effect. These findings offer preliminary evidence of exercise-induced “transfer effects” to the dietary domain, though many questions still remain about where to look for them.

In two complementary multi-studies (Blanchfield et al., 2014; Pageaux et al., 2015) Marcora and colleagues tested the effects of mental fatigue on submaximal and maximal exercise performance and ratings of perceived exertion (RPE; Pageaux et al., 2015), as well as the effects of subliminally-primed cues, i.e., visual faces and action words, on endurance and RPE (Blanchfield et al., 2014). Contrary to hypotheses, Pageaux et al. (2015) found that participants' maximal exertion was not impacted by a 30-min Stroop task intended to induce central and peripheral fatigue. Performance on submaximal exercise was altered, and this was also associated with higher RPE. Blanchfield et al. (2014) showed that persistence increased and RPE was lower when they primed participants with happy faces (experiment 1) and action words (experiment 2). This series of studies underscore the influence of conscious and subconscious motives on the self-regulation of aerobic training.

Each paper in the topic offers unique insights into the rapidly growing field that we know as exercise neuroscience. Important future directions include examination of activity/brain health relationships in medical populations, and deeper understanding of mechanisms linking brain and behavior in the context of exercise.

## Author contributions

Both authors have made substantial contributions to this work at all stages, from the conception and drafting to the final revisions. We have each contributed important intellectual content, approved the final version, and agree to be accountable for all aspects of the work.

## Funding

The first author is currently funded by a grant from the National Heart, Lung, and Blood Institute (Grant # 5R21HL113410-02) as well as via three internal grants from the University of Illinois at Urbana-Champaign's Center on Health, Aging, and Disability, and Campus Research Board.

### Conflict of interest statement

The authors declare that the research was conducted in the absence of any commercial or financial relationships that could be construed as a potential conflict of interest.
